# Association of adiposity with morbidity in Finnish adults: A register-based follow-up study

**DOI:** 10.1177/14034948221148053

**Published:** 2023-03-14

**Authors:** Päivi Mäki, Kennet Harald, Jaana Lindström, Satu Männistö, Tiina Laatikainen

**Affiliations:** 1 Finnish Institute for Health and Welfare, Finland; 2 Institute of Public Health and Clinical Nutrition, University of Eastern Finland, Finland; 3 Joint Municipal Authority for North Karelia Social and Health Services (Siun sote), Finland

**Keywords:** Overweight, obesity, adiposity, morbidity, noncommunicable diseases, follow-up study

## Abstract

**Aims:**

The aims of this study were to update risk estimates of obesity related co-morbidities and to provide evidence of the importance of obesity prevention to decision makers.

**Methods:**

The study included 25- to 74-year-old participants (*N*=22,977) of the National FINRISK Studies in 1997, 2002 and 2007. Body mass index was calculated from measured weight and height at baseline. Data on morbidity were ascertained via linkage to the National Hospital Discharge Register, the Cancer Register and the records of the Social Insurance Institution of Finland until the end of year 2018. The Cox proportional hazards model was used to estimate associations between weight status and the risk of the end-point diseases during follow-up, with adjustment for age and smoking.

**Results:**

At baseline, 31% of participants had at least one of the investigated diseases. Overweight, obesity and severe obesity were associated with type 2 diabetes, gout, gallbladder diseases and knee and hip osteoarthritis during the follow-up in both men and women. The risk of coronary heart disease was increased in men who were overweight, obese and severely obese and in women who were obese and severely obese. Risk of asthma was increased only among women who were obese and severely obese. No associations were found between obesity and breast, prostate or colorectal cancer.

**Conclusions:**

**The study showed a strong relationship between excess body weight and the prevalence and incidence of several diseases. Obesity prevention is essential to reduce disease burden in the future.**

## Introduction

The prevalence of people living with overweight and obesity have been rising over the last decades worldwide as well as in Finland [1]. In 2017, 72% of Finnish working-aged men and 63% of women were overweight (including obesity; body mass index (BMI) ⩾25 kg/m^2^), and 26% of men and 28% of women were obese (BMI ⩾30 kg/m^2^) [2].

Overweight and obesity lead to many negative effects on health. Previous findings have shown a strong association between overweight, obesity and the risk of type 2 diabetes [3–6]. A systematic review and meta-analyses of five Mendelian randomisation studies concluded that men and women who were obese had an increased risk of type 2 diabetes (odds ratio (OR)=1.67; 95% confidence interval (CI) 1.30–2.14) and coronary artery disease (OR=1.20; 95% CI 1.02–1.41) compared to men and women who were a healthy weight [5]. A review and meta-analysis including 87 studies reported statistically significant associations for obesity with type 2 diabetes, cardiovascular diseases, asthma, gallbladder disease, osteoarthritis, chronic back pain and kidney, colorectal, ovarian, pancreatic, uterine/endometrial cancer and post-menopausal breast cancer [4]. Furthermore, obesity has been associated with increased mortality [7–9] and decreased life expectancy [8,10].

However, the association between obesity and morbidity is complex due to the interrelationships between genetic, behavioural, socio-economic and environmental influences [11,12]. It has also been indicated that the risk of morbidity and mortality associated with overweight and obesity varies between studies and subgroups (e.g. by age, sex and race) [4,7,9]. This variation could be due to different study designs, for example study size, methods, measured or self-reported anthropometric values and outcomes, which makes comparison between studies and interpretation of results difficult.

There is a need for comparable risk estimates that are based on large population-based cohorts with objectively measured weight statuses and adequate follow-ups, as well as consideration of both prevalent and incident morbidity.

### Aims

The aim of this study was to examine the association of overweight, obesity and severe obesity with prevalence and incidence of type 2 diabetes, coronary heart disease, asthma, knee or hip osteoarthritis, gallbladder diseases, gout, colorectal, breast and prostate cancer in three Finnish population-based cohorts with a measured baseline obesity status and with register-based follow-up outcomes.

## Methods

### Data source and sample

The data were derived from the population-based National FINRISK Study [13]. FINRISK is a series of health examination surveys, which have been conducted every five years since 1972 by the Finnish Institute for Health and Welfare (THL). For each cohort, a stratified random sample was drawn from the population register.

The current study consisted of 25- to 74-year-old participants (Supplemental Table I) from the FINRISK 1997, 2002 and 2007 cohorts, carried out in five large study areas (the cities of Helsinki and Vantaa in Southern Finland, the provinces of North Karelia and North Savo in Eastern Finland, the Turku-Loimaa area in Southwestern Finland and the Oulu province in Northern Finland) [13]. The participants received a letter inviting them to a health examination and a questionnaire to be filled in beforehand at home [13]. The questionnaire included information on the participant’s socio-demographic factors (e.g. education), health behaviour (e.g. smoking) and medical history. The health examination included anthropometric measures and blood samples.

The study protocol and all procedures involving participants were approved by the Ethics Committee of the National Public Health Institute (FINRISK 1997) and the Ethics Committee of the Helsinki and Uusimaa Hospital District (FINRISK 2002 and 2007). Written informed consent was obtained from all participants, and data protection, confidentiality and pseudonymity were assured [13].

### Baseline assessment of weight and height

The participants’ heights and weights were measured by trained study nurses [13]. Weight was measured with a beam-balanced scale to the nearest 100 g without shoes and wearing only light clothing. Height was measured with a stadiometer to the nearest 0.5 cm (1997) and 0.1 cm (2002 onwards). BMI was calculated as weight in kilograms divided by height in meters squared (kg/m^2^). The participants’ weight status was classified by BMI using cut-off points defined by the World Health Organization [14]: healthy weight, including underweight (BMI <25 kg/m^2^), overweight (BMI 25–29.9 kg/m^2^), obesity (BMI 30–34.9 kg/m^2^) and severe obesity (BMI ⩾35 kg/m^2^).

### Outcome ascertainment

Information on the onset of the selected diseases (type 2 diabetes, coronary heart disease, asthma, knee or hip osteoarthritis, gallbladder diseases, gout and colorectal, breast and prostate cancer) at baseline and during follow-up was obtained from the National Hospital Discharge Register, the Cancer Register and the records of the Social Insurance Institution of Finland using the dates of the first record indicating diagnosis of the studied diseases (Supplemental Table II). The combined study cohort was followed up until the end of 2018, except for reimbursable medicines for which the follow-up concluded at the end of 2017.

### Statistical analysis

All analyses were done separately for men and women, and by weight status and age group at baseline. For analysis, the participants were divided into age groups according to age at baseline as follows: asthma 25–54 years of age, prostate and post-menopausal breast cancer 50–74 years of age and other investigated diseases 35–74 years of age. This was done based on adequate incidence of new cases in disease groups and to avoid bias caused by incidence of diseases where differential diagnoses is difficult (i.e. chronic obstructive pulmonary disease).

Due to the small number of cancer cases, obesity and severe obesity were combined in the analyses. Participants with end-point disease at baseline were excluded from the follow-up analysis of that disease. Associations between weight status at baseline and incidence of end-point diseases were tested using the Cox proportional hazards model. In analyses, adjustments for age, education, smoking and alcohol were carried out. In the final models, hazard ratios (HR) and 95% CIs were adjusted for age and smoking status, as the other factors did not affect the results. The proportional hazards assumption was tested using the Schoenfeld residuals, and the assumption was not violated.

The analyses were conducted in R v3.6 [15], and Cox models were produced using the package survival [16].

## Results

In 1997, 2002 and 2007, a total of 22,977 individuals were surveyed, of whom 48% were men and 52% women. The prevalence of overweight was 48% and 34%, obesity 17% and 15%, and severe obesity 4% and 7% in men and women, respectively (Supplemental Table I). At baseline, 31% of the participants had at least one of the end-point diseases. Overweight, obesity and severe obesity were associated with most of the end-point diseases at baseline as well as with the incidence of new cases in both sexes (Supplemental Figures 1–8). The association was the strongest with type 2 diabetes: the prevalence of type 2 diabetes was 2.1%, 4.5%, 9.8% and 15.7% in men and 1.5%, 3.7%, 6.9% and 10.5% in women with a healthy weight, overweight, obese and severely obese, respectively (Supplemental Figure 1).

In a multivariable model, a statistically significant upward trend in incidence was observed between overweight, obesity and severe obesity at baseline and type 2 diabetes, with the highest risk in men and women with severe obesity (Table I). HRs for type 2 diabetes in men who were overweight, obese and severely obese were 2.95 (95% CI 2.28–3.83), 7.25 (95% CI 5.56–9.47) and 15.45 (95% CI 11.40–20.94), respectively, compared to men with a healthy weight. The corresponding HRs for women were 2.45 (95% CI 1.92–3.13), 5.68 (95% CI 4.42–7.31) and 11.50 (95% CI 8.84–14.94), respectively. The risk of gout, knee or hip osteoarthritis and gallbladder diseases also increased in men and women who were overweight, obese and severely obese compared to those who were a healthy weight (Table I).

**Table I.** Cases during the follow-up period and hazard ratios for morbidity among men and women by weight status at baseline.

	Men		Women	
	n (%)	HR (95% CI)	n (%)	HR (95% CI)
Type 2 diabetes^a,b,c^				
Healthy weight	114	(ref.)	138	(ref.)
Overweight^d^	598	2.95 (2.28–3.83)	339	2.45 (1.92–3.13)
Obesity^d^	459	7.25 (5.56–9.47)	332	5.68 (4.42–7.31)
Severe obesity^d^	178	15.45 (11.40–20.94)	258	11.50 (8.84–14.94)
Coronary heart disease^a,b,c^				
Healthy weight	306	(ref.)	209	(ref.)
Overweight^d^	758	1.20 (1.01–1.43)	275	0.94 (0.76–1.17)
Obesity^d^	306	1.33 (1.08–1.64)	174	1.36 (1.07–1.73)
Severe obesity^d^	86	1.80 (1.33–2.44)	86	1.41 (1.04–1.91)
Asthma^a,b,e^				
Healthy weight	285	(ref.)	727	(ref.)
Overweight^d^	433	1.17 (0.97–1.41)	421	1.12 (0.97–1.31)
Obesity^d^	132	1.20 (0.92–1.56)	165	1.27 (1.03–1.56)
Severe obesity^d^	41	1.34 (0.92–1.96)	92	1.53 (1.15–2.04)
Knee and hip osteoarthritis^a,b,c^				
Healthy weight	143	(ref.)	235	(ref.)
Overweight^d^	422	1.59 (1.25–2.01)	476	2.17 (1.79–2.63)
Obesity^d^	213	2.45 (1.88–3.19)	290	3.23 (2.62–3.99)
Severe obesity^d^	63	2.98 (2.07–4.30)	160	3.99 (3.13–5.09)
Gallbladder diseases^a,b,c^				
Healthy weight	66	(ref.)	113	(ref.)
Overweight^d^	192	1.62 (1.11–2.37)	209	2.40 (1.81–3.19)
Obesity^d^	90	1.98 (1.30–3.01)	115	3.11 (2.26–4.29)
Severe obesity^d^	27	2.38 (1.38–4.09)	64	4.18 (2.85–6.13)
Gout^a,b,c^				
Healthy weight	62	(ref.)	31	(ref.)
Overweight^d^	285	2.54 (1.77–3.64)	100	3.17 (1.93–5.20)
Obesity^d^	189	5.39 (3.68–7.91)	79	5.11 (3.07–8.53)
Severe obesity^d^	71	9.33 (5.99–14.53)	78	11.17 (6.81–18.31)
Colorectal cancer^b,c,g^				
Healthy weight	20	(ref.)	32	(ref.)
Overweight^d^	72	1.40. (0.75–2.63)	40	0.76 (0.44–1.32)
Obesity/severe obesity^d^	38	1.99 (0.98–4.05)	39	1.22 (0.70–2.14)
Breast cancer^b,f,g^				
Healthy weight			89	(ref.)
Overweight^d^			98	0.72 (0.50–1.02)
Obesity/severe obesity^d^			77	0.77 (0.53–1.12)
Prostate cancer^b,f,g^				
Healthy weight	104	(ref.)		
Overweight^d^	247	0.91 (0.69–1.21)		
Obesity/severe obesity^d^	111	0.80 (0.57–1.11)		
All three cancers combined^b,c,g^				
Healthy weight	116	(ref.)	109	(ref.)
Overweight^d^	305	0.99 (0.76–1.30)	131	0.73 (0.54–1.00)
Obesity/severe obesity^d^	137	0.89 (0.65–1.21)	109	0.82 (0.60–1.13)

a Median follow-up time 16.8 years.

b Adjusted by age and smoking status.

c Age at baseline 35–74 years.

d Overweight, obesity and severe obesity were classified according to body mass index (BMI kg/m^2^) at baseline. Overweight: BMI 25–29.9 kg/m^2^; obesity: BMI 30–34.9 kg/m^2^; severe obesity: BMI ≥35 kg/m^2^.

e Age at baseline 25–54 years.

f Age at baseline 50–74 years.

g Median follow-up time 15.8 years.

CI: confidence interval; HR: hazard ratio.

Being overweight, obese and severely obese were statistically significantly associated with the incidence of coronary heart diseases in men, while in women, being obese and severely obese increased the risk compared to women with a healthy weight. The risk of asthma increased only in women who were obese or severely obese. However, men and women who were overweight or obese (including severely obese) did not have an increased risk of colorectal, breast or prostate cancer during follow-up compared to participants with a healthy weight at baseline.

## Discussion

We found a strong relationship between excess body weight and prevalence and incidence of type 2 diabetes, coronary heart diseases, gout, knee or hip osteoarthritis and gallbladder diseases in both men and women. The association was strongest with type 2 diabetes and weakest with coronary heart disease. In addition, the risk of coronary heart disease increased in all obesity categories in men, but in women only with obesity and severe obesity. No association was found between overweight or obesity and the risk of asthma (men) and colorectal, breast or prostate cancer. A systematic review that included 27 meta-analyses and 15 cohort studies also concluded that both overweight and obesity were associated with increased disease-specific morbidity in some, but not all, diseases [9]. Furthermore, previous studies have shown that obesity confers a higher risk than overweight [9], and the risk of diseases varies between different age groups and sexes [9,17,18].

Like others, we found that overweight and obesity associated most strongly with type 2 diabetes, and the risk of type 2 diabetes increases with increasing BMI [4,5,7,19,20]. A review of 89 studies in Europe or North America, Australia or New Zealand with a follow-up of 12.5 years reported that men who were obese had a sevenfold higher risk and women who were obese had a 12-fold higher risk of developing type 2 diabetes compared to men and women who were a healthy weight [4]. As concluded in a previous review [21], we also found that the risk of type 2 diabetes was higher in men than in women during follow-up in each obesity category. Sex differences arise from many factors and mechanisms, such as genetic effects and epigenetic mechanisms, biological (e.g. body composition, sex hormones, metabolism), nutritional factors and sedentary lifestyle [21]. For example, men are more prone to abdominal obesity than women [22]. In addition, psychosocial factors (e.g. socio-economic status, and work-related stress) or attitudes towards treatment and prevention may explain sex differences [21].

In our study, the risk of coronary heart disease was increased in all male obesity categories, and in women with obesity and severely obese compared to participants with a healthy weight. Many previous studies have shown that overweight and obesity increase the risk of cardiovascular diseases, especially coronary heart disease [20,23,24]. Obesity can increase morbidity both directly and indirectly mediated by co-existing risk factors of coronary heart disease such as insulin resistance, hyperglycaemia, hypertension and dyslipidaemia [23]. A meta-analysis, including 21 cohorts and more than 300,000 healthy participants, concluded that even for those who were moderately overweight, there was a significant increased risk of coronary heart disease independent of traditional risk factors, although confounding (e.g. dietary) factors could not be completely ruled out [20]. A five-unit increase in BMI was associated with a 29% increase in risk of coronary heart disease, and after additional adjustment for blood pressure and cholesterol concentration, the increased risk was 16%.

We found that the risk of coronary heart disease was increased in men who were overweight, obese and severely obese but only in women who were obese and severely obese. In contrast, in the Framingham Heart Study, with participants aged 35–75 years, the age-adjusted risk of cardiovascular disease was similar in both men and women who were overweight (men 1.21, 95% CI 1.05–1.40; women 1.20, 95% CI 1.03–1.41). The risk of women with obesity was slightly higher than in men (men 1.46, 95% CI 1.20–1.77; women 1.64, 95% CI 1.37–1.98) [25]. One possible explanation for the differences between the results may be different follow-up periods.

In our study, the risk of asthma increased in women who were obese or severely obese, but not in men with excess body weight. Our results are consistent with previous findings that asthma is more frequently diagnosed in women with obesity [26,27]. In line with our study, a Canadian survey with 4266 men and 4883 women aged 20–64 years showed that baseline BMI was a significant predictor for the incidence of asthma in women (OR=1.9; 95% CI 1.1–3.4) during a two-year follow-up period, but not in men [26].

Like others, we also found an increased risk of knee or hip osteoarthritis in men and women in all obesity categories compared to men and women with a healthy weight [28,29]. As in previous studies, we found a strong association between overweight, obesity, severe obesity and gallbladder diseases, including cholelithiasis and cholecystitis, and between overweight, obesity, severe obesity and gout in both men and women [4,30,31].

High BMI has been recognised as an important risk factor for several types of cancers [32]. We found no statistically significant association between overweight or obesity (including severe obesity) and the risk of colorectal, post-menopausal breast or prostate cancer. One explanation for our results may be the relatively small number of cancer cases, which reduced statistical power.

This study has some strengths and limitations. The main strengths of our study were large population-based cohorts with measured height and weight and diagnosed end-point diseases obtained from national health-care records with proven validity [33]. Moreover, the study design was similar in different cohorts, and the follow-up time was long. Comprehensive register data and survey data enabled the health status of the participants to be identified at baseline and during follow-up. However, there are also some limitations to the study. First, weight was measured only at baseline, and changes in weight status might have occurred, typically an increase in body weight but sometimes a reduction. In Finland, the mean annual increase in weight among adults has been around 0.3 kg in both sexes [34]. Our risk estimates are thus likely to reflect the risk of populations with slow, continuous weight gain. Second, the follow-up periods of the 1997, 2002 and 2007 FINRISK cohorts were different (minimum 11 years, maximum 21 years) because the follow-up ended by 2018, except for reimbursable medicines, for which the follow-up concluded at the end of 2017. However, as we used the Cox logistic regression model, the actual time to event was used in the hazard model. In addition, as a person may have type 2 diabetes without clear symptoms for years before diagnosis, there were probably undiagnosed cases, which may have attenuated our findings. Furthermore, in this study, analyses were adjusted for age and smoking status. It is possible that in some diseases there are other important confounding factors, the effect of which we were unable to account for and which may have influenced the results.

The prevalence of overweight and obesity has increased in Finland and globally, and hence our findings have important public health implications. The rising trend in obesity has received a lot of attention in Finland and has led to health policy activities such as a sweets and ice cream excise tax experiment and the establishment of a national obesity programme. Unfortunately, these measures have not been sufficient to halt the progression of obesity, and it has been estimated that the proportion of people with obesity will continue to increase [35]. There is a need for continued focus on the primary prevention of obesity and the maintenance of a healthy weight to promote healthy life years and to prevent a large disease burden in the future. Because the association between obesity and morbidity is complex, multiple society-level actions and good collaboration between different stakeholders are needed [36].

## Conclusions

The study, with comparable risk estimates and objectively measured weight statuses and adequate follow-ups with registry confirmed diagnoses, showed a strong relationship between excess body weight and the prevalence and incidence of many diseases. The prevention of obesity is essential to reduce disease burden in the future.

## Supplemental Material

sj-docx-1-sjp-10.1177_14034948221148053 – Supplemental material for Association of adiposity with morbidity in Finnish adults: A register-based follow-up study Supplemental material, sj-docx-1-sjp-10.1177_14034948221148053 for Association of adiposity with morbidity in Finnish adults: A register-based follow-up study by Päivi Mäki, Kennet Harald, Jaana Lindström, Satu Männistö and Tiina Laatikainen in Scandinavian Journal of Public Health

sj-docx-2-sjp-10.1177_14034948221148053 – Supplemental material for Association of adiposity with morbidity in Finnish adults: A register-based follow-up study Supplemental material, sj-docx-2-sjp-10.1177_14034948221148053 for Association of adiposity with morbidity in Finnish adults: A register-based follow-up study by Päivi Mäki, Kennet Harald, Jaana Lindström, Satu Männistö and Tiina Laatikainen in Scandinavian Journal of Public Health

sj-docx-3-sjp-10.1177_14034948221148053 – Supplemental material for Association of adiposity with morbidity in Finnish adults: A register-based follow-up study Supplemental material, sj-docx-3-sjp-10.1177_14034948221148053 for Association of adiposity with morbidity in Finnish adults: A register-based follow-up study by Päivi Mäki, Kennet Harald, Jaana Lindström, Satu Männistö and Tiina Laatikainen in Scandinavian Journal of Public Health

sj-docx-4-sjp-10.1177_14034948221148053 – Supplemental material for Association of adiposity with morbidity in Finnish adults: A register-based follow-up study Supplemental material, sj-docx-4-sjp-10.1177_14034948221148053 for Association of adiposity with morbidity in Finnish adults: A register-based follow-up study by Päivi Mäki, Kennet Harald, Jaana Lindström, Satu Männistö and Tiina Laatikainen in Scandinavian Journal of Public Health
