# Supplementary material for: Association of adiposity with morbidity in Finnish adults: A register-based follow-up study
Source: Scand J Public Health. 2023 Mar 14;52(4):461–7. doi: 10.1177/14034948221148053 (PMC11179310; doi:10.1177/14034948221148053)
Supplement: sj-docx-1-sjp-10.1177_14034948221148053 – Supplemental material for Association of adiposity with morbidity in Finnish adults: A register-based follow-up study [file sj-docx-1-sjp-10.1177_14034948221148053.docx]

**Supplemental material for “Association of adiposity with morbidity in Finnish adults: a register-based follow-up study” by Päivi Mäki, Kennet Harald, Jaana Lindström, Satu Männistö, Tiina Laatikainen**

Supplementary table 1. Prevalence of overweight, obesity and severe obesity and prevalence of diseases by age group, gender, and weight status at baseline

Supplementary table 2. ICD codes and drug reimbursement right codes for each end point disease at baseline and during follow-up

Supplementary figure 1. Type 2 diabetes (T2D) prevalence at baseline and incidence during the follow-up by weight status at baseline in men (a) and in women (b)

Supplementary figure 2. Coronary heart disease prevalence at baseline and incidence during the follow-up by weight status at baseline in men (a) and in women (b)

Supplementary figure 3. Asthma prevalence at baseline and incidence during the follow-up by weight status at baseline in men (a) and women (b)

Supplementary figure 4. Knee or hip osteoarthritis prevalence at baseline and incidence during the follow-up by weight status at baseline in men (a) and women (b)

Supplementary figure 5. Gallbladder disease prevalence at baseline and incidence during the follow-up by weight status at baseline in men (a) and women (b)

Supplementary figure 6. Gout prevalence at baseline and incidence during the follow-up by weight status at baseline in men (a) and women (b)

Supplementary figure 7. Colorectal cancer prevalence at baseline and incidence during the followup by weight status at baseline in men (a) and women (b)

Supplementary figure 8. Prostate cancer prevalence at baseline and incidence during the follow-up by weight status at baseline (men only)

Supplementary figure 9. Breast cancer prevalence at baseline and incidence during the follow-up by weight status at baseline (women only)
