# Supplementary material for: Association of adiposity with morbidity in Finnish adults: A register-based follow-up study
Source: Scand J Public Health. 2023 Mar 14;52(4):461–7. doi: 10.1177/14034948221148053 (PMC11179310; doi:10.1177/14034948221148053)
Supplement: sj-docx-2-sjp-10.1177_14034948221148053 – Supplemental material for Association of adiposity with morbidity in Finnish adults: A register-based follow-up study [file sj-docx-2-sjp-10.1177_14034948221148053.docx]

p for trend*=0.0001

1. Men

p for trend*=0.0001

1. Women

Supplementary figure 1. Type 2 diabetes (T2D) prevalence at baseline and incidence during the follow-up by weight status at baseline in men (a) and in women (b).
Age at the baseline 35-74 years. Median follow-up time 16,8 years.
*Adjusted for age and smoking.

p for trend*=0.0001

1. Men

p for trend*=0.0001

1. Women

Supplementary figure 2. Coronary heart disease prevalence at baseline and incidence during the follow-up by weight status at baseline in men (a) and in women (b).
Age at the baseline 35-74 years. Median follow-up time 16,8 years.
*Adjusted for age and smoking.

p for trend*=0.001

1. Men

p for trend*=0.0001

1. Women

Supplementary figure 3. Asthma prevalence at baseline and incidence during the follow-up by weight status at baseline in men (a) and women (b).
Age at the baseline 25-54 years. Median follow-up time 16,8 years.
*Adjusted for age and smoking.

p for trend*=0.0001

1. Men

p for trend*=0.0001

1. Women

Supplementary figure 4. Knee or hip osteoarthritis prevalence at baseline and incidence during the follow-up by weight status at baseline in men (a) and women (b).
Age at the baseline 35-74 years. Median follow-up time 16,8 years.
*Adjusted for age and smoking.

p for trend*=0.0001

1. Men

p for trend*=0.0001

1. Women

Supplementary figure 5. Gallbladder disease prevalence at baseline and incidence during the follow-up by weight status at baseline in men (a) and women (b).
Age at the baseline 35-74 years. Median follow-up time 16,8 years.
*Adjusted for age and smoking.

p for trend*=0.0001

1. Men

p for trend*=0.0001

1. Women

Supplementary figure 6. Gout prevalence at baseline and incidence during the follow-up by weight status at baseline in men (a) and women (b).
Age at the baseline 35-74 years. Median follow-up time 16,8 years.
*Adjusted for age and smoking.

p for trend*=0,0510

1. Men

p for trend*=0,0894

1. Women

Supplementary figure 7. Colorectal cancer prevalence at baseline and incidence during the follow-up by weight status at baseline in men (a) and women (b).
Age at the baseline 35-74 years. Median follow-up time 15,8 years.
*Adjusted for age and smoking.

p for trend*=0,2602

Supplementary figure 8. Prostate cancer prevalence at baseline and incidence during the follow-up by weight status at baseline (men only).
Age at the baseline 35-74 years. Median follow-up time 15,8 years.
*Adjusted for age and smoking.

p for trend*=0,0766

Supplementary figure 9. Breast cancer prevalence at baseline and incidence during the follow-up by weight status at baseline (women only).
Age at the baseline 50-74 years. Median follow-up time 15,8 years.
*Adjusted for age and smoking.
