# Supplementary material for: Association of adiposity with morbidity in Finnish adults: A register-based follow-up study
Source: Scand J Public Health. 2023 Mar 14;52(4):461–7. doi: 10.1177/14034948221148053 (PMC11179310; doi:10.1177/14034948221148053)
Supplement: sj-docx-3-sjp-10.1177_14034948221148053 – Supplemental material for Association of adiposity with morbidity in Finnish adults: A register-based follow-up study [file sj-docx-3-sjp-10.1177_14034948221148053.docx]

Supplementary table 1. Prevalence of overweight, obesity and severe obesity and prevalence of diseases by age group, gender, and weight status at baseline

|  | **Men (N=10981)** | | | | | | | | | | | | **Women (N=11996)** | | | | | | |
| --- | --- | --- | --- | --- | --- | --- | --- | --- | --- | --- | --- | --- | --- | --- | --- | --- | --- | --- | --- |
|  | Normal weight  N (%) | | Overweight  N (%) | | Obesity  N (%) | | | Severe obesity  N (%) | | | Total  N (%) | | Normal weight  N (%) | Overweight  N (%) | Obesity  N (%) | | Severe obesity  N (%) | Total  N (%) | |
| **Age at baseline 25-75** | **3355 (30.6)** | | **5311 (48.4)** | | **1828 (16.6)** | | | **487 (4.4)** | | | **10981 (100)** | | **5378 (44,8)** | **4031 (33,6)** | **1734 (14,5)** | | **85 (7,1)** | **11996 (100)** | |
| **Age at baseline 35–74** | **2439 (26,8)** | | **4591 (50,4)** | | **1636 (18,0)** | | | **446 (4,9)** | | | **9112 (100)** | | **3765 (39,2)** | **3476 (36,2)** | **1590 (16,6)** | | **768 (8,0)** | **9599 (100)** | |
| Type 2 diabetes | 51 (2,1) | | 207 (4,5) | | | 161 (9,8) | | | 70 (15,7) | | 489 (5,4) | | 55 (1,5) | 128 (3,7) | | 109 (6,9) | 81 (10,5) | | 373 (3,9) |
| Coronary heart disease | 141 (5,8) | | 443 (9,6) | | | 205 (12,5) | | | 75 (16,8) | | 864 (9,5) | | 75 (2,0) | 146 (4,2) | | 127 (8,0) | 57 (7,4) | | 405 (4,2) |
| Knee or hip osteoarthritis | 58 (2,4) | | 197 (4,3) | | | 131 (8,0) | | | 38 (8,5) | | 424 (4,7) | | 76 (2,0) | 149 (4,3) | | 138 (8,7) | 95 (12,4) | | 458 (4,8) |
| Gallbladder diseases | 59 (2,4) | | 117 (2,5) | | | 75 (4,6) | | | 19 (4,3) | | 270 (3,0) | | 162 (4,3) | 239 (6,9) | | 168 (10,6) | 127 (16,5) | | 696 (7,3) |
| Gout | 29 (1,2) | | 102 (2,2) | | | 77 (4,7) | | | 33 (7,4) | | 241 (2,6) | | 10 (0.3) | 15 (0,4) | | 24 (1,5) | 28 (3,6) | | 77 (0,8) |
| Colorectal cancer | 7 (0,3) | | 13 (0,3) | | | 8 (0,5) | | | 3 (0,7) | | 31 (0,3) | | 7 (0,2) | 6 (0,2) | | 3 (0,2) | 6 (0,8) | | 22 (0,2) |
| **Age at baseline 25–54** | **2346 (36,5)** | | **2963 (46,1)** | | **893 (13,9)** | | | **224 (3,5)** | | | **6426 (100)** | | **4218 (54,9)** | **2250 (29,3)** | **804 (10,5)** | | **415 (5,4)** | **7687 (100)** | |
| Asthma | 135 (5,6) | | 132 (4,5) | | 52 (5,8) | | | 20 (8,9) | | | 339 (5,3) | | 288 (6,8) | 182 (8,1) | 85 (10,6) | | 58 (13,9) | 613 (8,0) | |
| **Age at baseline 50–74** | **1337 (23,3)** | | **2923 (50,9)** | | **1161 (20,2)** | | | **320 (5,6)** | | | **5741 (100)** | | **1663 (29,4)** | **2281 (40,4)** | **1150 (20,3)** | | **558 (9,9)** | **5652 (100)** | |
| Breast cancer | |  | |  | | |  | | |  | |  | 58 (3,5) | 61 (2,7) | 36 (3,1) | | 14 (2,5) | | 169 (3,0) |
| Prostate cancer | | 20 (1,5) | | 39 (1,3) | | | 15 (1,3) | | | 0 (0) | | 74 (1,3) |  |  |  | |  | |  |

^1^ Normal weight, overweight, obesity and severe obesity were classified according to the body mass index (BMI kg/m^2^) at the baseline. Normal weight (BMI < 25 kg/m^2^), overweight (BMI 25-29.9 kg/m^2^), obesity (BMI 30-34.9 kg/m^2^) and severe obesity (BMI >35 kg/m^2^).
