# Supplementary material for: Association of adiposity with morbidity in Finnish adults: A register-based follow-up study
Source: Scand J Public Health. 2023 Mar 14;52(4):461–7. doi: 10.1177/14034948221148053 (PMC11179310; doi:10.1177/14034948221148053)
Supplement: sj-docx-4-sjp-10.1177_14034948221148053 – Supplemental material for Association of adiposity with morbidity in Finnish adults: A register-based follow-up study [file sj-docx-4-sjp-10.1177_14034948221148053.docx]

Supplementary table 2. ICD codes and drug reimbursement right codes for each end point disease at baseline and during follow-up

| Disease | ICD-8 | ICD-9 | ICD-10 | ATC codes of medicines | Drug reimbursement right codes |
| --- | --- | --- | --- | --- | --- |
| Type 2 diabetes | 250 | 250 | At baseline E10-14  During follow up E11 | At baseline A10  During follow up A10B | 103 |
| Coronary heart disease | 410-414 | 410-414 | I20-I25 | - | 206 |
| Asthma | 493 | 493 (not 4939C) | J45, J46 | R03AK06, R03AK07, R03AK08, R03AK10, R03AK11, R03AL08, R03AL09, R03BA01, R03BA02, R03BA05, R03BA07, R03BA08, R03DC01, R03DC03. If there were only above-mentioned medicine codes without IDC-10 codes J45 or J46, those who have had ICD-10 codes J40, J41, J42, J43, J44, or J47 before medicines were excluded. | 203 |
| Knee and hip osteoarthritis | 715 | 715 | M15-M19 | - | - |
| Gout | 274 | 274 | At baseline M10  During follow up M10.0, M10.4, M10.9 | M04 | 212 |
| Cholelithiasis diseases | 574, 575.0, 575.1 | 574, 575.0, 575.1 | K80, K81 | - | - |
| Breast cancer | 174 | 174-175 | C50 | - | - |
| Prostate cancer | 185 | 185 | C61 | - | - |
| Colorectal cancer | 153, 154.0-154.2 | 153, 154.0-154.8 | C18-C21 | - | - |
